# Efficacy of *Escherichia coli* Nissle 1917 for the Prevention of Recurrent Urinary Tract Infections in Women: A Preliminary Controlled Prospective Study

**DOI:** 10.3390/clinpract16030062

**Published:** 2026-03-21

**Authors:** Filippo Murina, Cecilia Fochesato, Dario Recalcati, Valeria Savasi

**Affiliations:** 1 Lower Genital Tract Disease Unit, V. Buzzi Hospital, University of the Study of Milan, 20154 Milan, Italy; cecilia.fochesato@asst-fbf-sacco.it (C.F.); dario.recalcati@asst-fbf-sacco.it (D.R.); 2 Clinical Obstetric and Gynecological, V. Buzzi Hospital, ASST-FBF-Sacco, University of the Study of Milan, 20154 Milan, Italy; valeria.savasi@unimi.it

**Keywords:** urinary tract infections, probiotic, *E. coli*, prophylaxis, non-antimicrobial

## Abstract

Background/Objectives: More than 50% of adult women experience at least one urinary tract infection (UTI) during their lifetime, and approximately 25% develop recurrent UTIs (rUTIs), defined as ≥2 episodes within six months. Management of rUTI is challenging and often requires long-term, multimodal preventive strategies. *Escherichia coli* Nissle 1917 (EcN) is a non-pathogenic probiotic strain with demonstrated antagonistic activity against pathogenic enterobacteria. This study evaluated the efficacy and safety of EcN in preventing symptomatic recurrences in premenopausal women with rUTI. Methods: In this prospective observational study, 40 premenopausal women with rUTI were enrolled. Twenty patients received EcN prophylaxis (twice daily for four weeks, followed by once daily for eight weeks), while 20 patients received no prophylaxis and served as controls. Patients were followed for six months (three months of treatment and three months post-treatment). The primary outcome was the frequency of symptomatic rUTI episodes during follow-up. Results: Forty patients were analyzed (20 EcN; 20 controls). During the six-month observation period, 55% (11/20) of patients in the EcN group remained UTI-free compared with 35% (7/20) in the control group. Two patients (10%) in the EcN group experienced a single recurrence versus three (15%) in the control group. Recurrent episodes (≥2 UTIs) occurred in 35% (7/20) of EcN-treated patients compared with 50% (10/20) of controls. Overall, EcN prophylaxis was associated with a lower proportion of patients experiencing multiple recurrences. Conclusions: Prophylaxis with *E. coli* Nissle 1917 was associated with a reduced rate of recurrent UTIs compared with no prophylaxis in premenopausal women, supporting its potential role as a non-antibiotic preventive strategy in rUTI management.

## 1. Introduction

Urinary tract infections (UTIs) are one of the most frequent human diseases, whose high prevalence is a global health problem. Between the ages of 15 and 50 years, UTIs are virtually non-existent in men, while in women the prevalence may reach 3% [1]. Around 40–50% of women will experience more than one UTI during their lifetime. This situation is particularly frequent in young women due, in more than 80% of cases, to *Escherichia coli*. Recurrent UTI (rUTI) is defined as 2 or more UTIs in a 6-month period or 3 or more UTIs in 12 months [2]. Each episode has a considerable impact on the quality of life, as it is associated with approximately 6 days of persistence of symptoms. Proper diagnosis and effective therapy of rUTIs are crucial due to the considerable cost that these infections impose on both people and society. In recent years, increasing attention has been directed toward the role of the urinary microbiome and the so-called gut–bladder axis. Contrary to the long-held belief that urine is sterile, advanced molecular techniques have demonstrated that the bladder harbors a distinct microbial community [1,2]. Alterations in the composition and diversity of the urinary and intestinal microbiota have been associated with susceptibility to rUTIs. The gut is considered a primary reservoir of UPEC, and intestinal colonization often precedes ascending urinary infection. Disruption of microbial homeostasis in the gut–vaginal–bladder axis may therefore play a central role in recurrence pathogenesis [1,2].

After effective management of the acute UTI episode, preventive strategies are essential to diminish the frequency and morbidity of rUTIs. All guidelines concur that there is adequate evidence to recommend D-mannose and cranberry supplements for these individuals, however data supporting the use of probiotics and lactobacillus products are insufficient [3]. *E. coli* is the predominant bacteria responsible for uncomplicated urinary tract infections, accounting for around 85% of cases [4]. The infection of the bladder by Ur pathogenic *E. coli* (UPEC) has been thoroughly characterized. *Escherichia coli* infects the bladder through the urethra and adheres to the bladder epithelium, promoting bacterial colonization and invasion. Antimicrobials constitute the primary recommended treatment for rUTI [3]. Multiple solutions are suggested; nevertheless, it is concerning because UPEC is exhibiting increased resistance to numerous antimicrobials utilized for the treatment of UTIs. Global resistance to ciprofloxacin and other fluoroquinolones is growing rapidly warranting caution in their extensive use for treating UTIs [5,6].

The concerning rise in bacterial resistance to antibiotics represents a worldwide danger to future infection treatment and necessitates the development of alternate prevention and treatment models for rUTI. Despite growing interest in microbiome-targeted therapies, clinical data supporting the use of specific probiotic strains in rUTI prevention remain limited, particularly in well-defined populations such as premenopausal women [2]. Given the increasing burden of antimicrobial resistance and the need for effective non-antibiotic preventive strategies, further investigation of targeted probiotic interventions is warranted.

*Escherichia coli* Nissle 1917 (EcN) is a non-pathogenic probiotic strain that has been thoroughly investigated for its therapeutic effects on gastrointestinal diseases, including inflammatory bowel disease, infections, and metabolic disorders [7]. Its distinctive genetic composition, absence of traditional virulence factors, and multiple mechanisms of action—including antimicrobial peptide synthesis, competitive exclusion, and immunomodulation—distinguish it from harmful *E. coli* strains. EcN demonstrates metabolic plasticity, biofilm formation capability, and interact with host immunity, these characteristics are essential to its probiotic function [8]. EcN was isolated by Prof. Alfred Nissle from the excrement of a German soldier in 1917. This is a nonpathogenic Gram-negative strain that does not exhibit recognized virulence factors, including α-hemolysin and P-fimbrial adhesins [9]. Instead, this strain demonstrates significant antagonistic activity against many pathogenic enterobacteria. Our study aims to assess the efficacy of EcN in preventing rUTI in premenopausal women, with a focus on its effect on the frequency of symptomatic UTI recurrences.

## 2. Materials and Methods

### 2.1. Study Design

This single-center study was performed at the Lower Genital Tract Disease Unit, V. Buzzi Hospital in Milan, Italy (beginning of the research the 1 October 2024), and involved premenopausal women aged 18 to 50 years with an acute UTI and a history of uncomplicated rUTI, defined as three or more documented UTI episodes in the past year or two episodes in the last six months. The diagnosis of an acute urinary tract infection (UTI) was established based on the detection of ≥10^3^ colony-forming units (CFUs)/mL in clean voided midstream urine, in accordance with European Association of Urology guidelines [10], together with the presence of two or more lower urinary tract symptoms (LUTS): dysuria, nocturia, urgency, frequency, suprapubic pain, or hematuria. Patients were excluded if they were pregnant, breastfeeding, or attempting to conceive; exhibited symptoms of upper urinary tract infection and systemic inflammatory response; had a history of urinary tract anomalies, interstitial cystitis, or diabetes; had previously undergone antibiotic prophylaxis for urinary tract infections; or had received probiotic treatment or immunotherapy within two weeks prior to the study. All participants provided written informed consent prior to their involvement in the study. The ethics committee review board of V. Buzzi Hospital, Milan, Italy, granted approval for this study’s conduct. This study was performed in compliance with the Helsinki Declaration as amended in 2013.

### 2.2. Treatments and Procedures

The initial urinary tract infection was treated with Fosfomycin 3 g administered once daily for two consecutive days, according to EAU guidelines in case of recurrences, followed by the analysis of control urine samples 7–10 days after the end of therapy. Patients with fewer than 10^3^ CFU/mL and resolution of LUTS were classified as cured and were randomized in a 1:1 ratio into two groups for a 6-month prophylaxis phase.

Group 1 received oral EcN (Mutaflor^®^ Ardeypharm GmbH, Herdecke, Germany and EcN, Cadigroup, In Italy) at a dosage of 320 mg (2.5 × 10^9^ CFU per capsule), administered twice daily for four weeks, followed by once daily for eight weeks. Group 2, the control group, received no prophylactic treatment. A clean voided midstream urine sample for culture was obtained when a participant reported symptoms indicative of a UTI during prophylaxis. A criterion of 10^3^ CFU/mL or greater was employed to define recurrent infection. In instances of recurrence, prophylaxis was discontinued, and antibiotic therapy was initiated. Patients experiencing more than two recurrences were excluded from the study, whereas those with one or two recurrences were allowed to continue prophylaxis. Three visits were scheduled: the inclusion and initial visit (day 0), a control visit following the completion of treatment with EcN (day 90), and a final follow-up visit (day 180, three months post-therapy).

### 2.3. Endpoints

The primary endpoint of the study was the incidence of recurrent urinary tract infection (rUTI) during the six-month observation period (180 days), including both the treatment phase and the post-treatment follow-up.

Participants were categorized into three predefined response groups based on the number of documented recurrences during the study period:

− *Responders* were defined as patients who remained free from symptomatic UTI recurrence from baseline through day 180. Absence of recurrence was defined as no occurrence of urinary symptoms suggestive of infection (including suprapubic pain, urinary frequency, urgency, dysuria, or urethral discomfort) and no microbiologically confirmed UTI during the study period.
− *Partial responders* were defined as patients who experienced no more than one documented UTI recurrence during either the EcN treatment phase or the subsequent 90-day post-treatment follow-up.
− *Non-responders* were defined as patients who developed two or more recurrent UTI episodes during the treatment phase or within the 90-day follow-up period.

At the end of the 180-day study period, the proportion of participants who remained UTI-free was calculated. In addition, all adverse events potentially related to the intervention were systematically recorded and evaluated for severity and causality.

### 2.4. Statistical Analysis

Continuous variables were summarized using conventional descriptive statistics, such as mean and range, and comparisons were performed using Student’s *t*-test. Categorical variables were analyzed through the chi-square test. Statistical significance was defined as a *p*-value < 0.05. The sample size estimation was informed by previously reported data indicating an average of four UTI recurrences per year, with the study hypothesizing a reduction to 1.5 episodes in the EcN-treated cohort. On the basis of an alpha error of 0.05, a statistical power of 80%, and an assumed standard deviation of two recurrence events over a six-month interval, it was calculated that 20 participants per group (40 in total) would be necessary to detect a difference of 2.5 episodes.

## 3. Results

A total of 40 women participated in this study, with 20 using EcN and 20 serving as the control group without any prophylaxis. The baseline characteristics of the two groups were similar for most variables, including age, body mass index (BMI), length of menstrual cycles, use of hormonal contraceptives, and parity (see Table 1).

A mean of 4.38 ± 1.34 culture-positive UTIs had been encountered by each patient in the 12 months prior to EcN treatment, without significant difference among the two groups. During the acute phase of cystitis, the predominant isolated bacterium was *Escherichia coli*, accounting for 86.6% of cases. This was followed by Enterococcus faecalis at 12.2%, and Proteus mirabilis at 1.2%. No significant differences were observed among the groups regarding isolated microorganisms. All patients were declared free of urinary tract infection following Fosfomycin treatment and prior to the commencement of prophylaxis.

**Figure 1:**
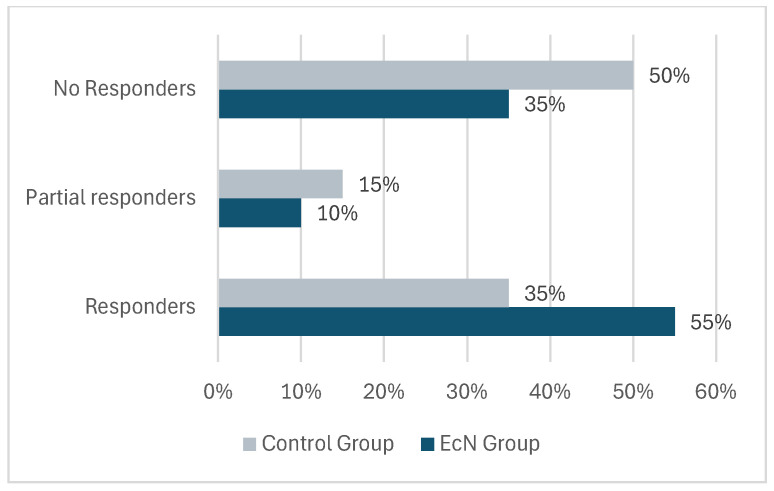
Rate of urinary tract infection relapses during the study. Responders: Patients UTI-free for 6 months; Partial responders: Patients with only one UTI episode. Non-responders: Patients with two or more UTI episodes.

The incidence of UTI episodes throughout the study was markedly greater in the control group than in the EcN Group (Figure 1).

In the six months following the initiation of EcN (three months of treatment plus three months of follow-up), 11 patients (55%) were UTI-free and therefore considered as responders, 2 patients (10%) reported only one UTI episode (partial responders), and the remaining 7 patients (35%) presented two or more UTIs (no responders). Instead, in the control group that did not receive any prophylaxis treatment, only 7 patients (35%) were classified as responders, while 3 patients (15%) and 10 patients (50%) were classified as partial or non-responders, respectively.

When looking at whether patient traits affected the reduction in UTIs with EcN, there were no significant differences related to age, BMI, regular sexual activity, menstrual periods, or use of hormonal contraceptives. No adverse events were reported during treatment, and no patients withdrew from the study.

## 4. Discussion

Our data indicate that EcN therapy is helpful in preventing recurrent UTIs. In fact, 55% of patients reported no UTIs in the six months following treatment, and 10% reported only one episode of UTI. This result was significantly greater than in a control group without any prophylaxis, in which only 35% were UTI-free after six months of follow-up. These promising outcomes indicate that EcN could serve as a viable alternative to traditional antibiotic prophylaxis, potentially reducing the reliance on antibiotics and lowering the risk of resistance. Various preventative strategies exist for reducing rUTIs, and different approaches can be integrated to enhance relapse reduction and enhance long-term antimicrobial-sparing approaches. Indeed, although antibiotic prophylaxis is supported by substantial evidence of its effectiveness, the possible risks associated with adverse events and the development of resistance warrant cautious consideration [1]. Cranberry and mannose are prevalent non-antimicrobial therapies utilized in clinical practice. Proanthocyanidins, naturally occurring compounds in cranberries, are believed to inhibit bacterial adherence to the bladder epithelium, while mannose may saturate *E. coli* Type 1 fimbriae, which are implicated in bacterial pathogenicity and biofilm formation, significant virulence factors in pathogenic *E. coli* strains [11,12]. The urinary microbiome is correlated with rUTIs [13]. Particularly, alterations leading to the depletion of typically protective Lactobacillus spp. appear to elevate the risk of urinary tract infections. The vaginal tract is believed to contribute to UTI pathogenesis by acting as a possible reservoir for pathogenic bacteria ascending from the gastrointestinal tract. Research indicates that women with recurrent urinary tract infections exhibit a reduced presence of lactobacilli and are more frequently colonized by vaginal *E. coli* [13]. Significant discrepancies in the support of non-antibiotic preventive treatments were noted among the guidelines. Although some trials provide effectiveness data on the use of mannose and cranberry for the prevention of rUTI, the evidence remains contradictory for making a definitive recommendation for these products [3]. Moreover, all guidelines concur that there is still inadequate evidence to support probiotics and Lactobacillus products in women with rUTI.

EcN has been extensively utilized as an adjuvant in the management of various intestinal disorders, including inflammatory bowel disease, irritable bowel syndrome, diarrhea, persistent constipation, and ulcerative colitis [14].

To our knowledge, this study is the first to specifically assess the effectiveness of EcN in the prevention of uncomplicated rUTI. The multifaceted effects of EcN on the pathophysiological mechanisms of rUTIs explain our positive results.

Numerous studies have indicated that the antibacterial efficacy of this strain is mostly attributed to a direct antimicrobial impact resulting from the secretion of two particular bacteriocins: microcin H47 and microcin M [8]. The EcN strain interacts extensively with the epithelium and the mucosal immune system, eliciting immunomodulatory and anti-inflammatory effects, while also enhancing the epithelial permeability barrier and fostering beneficial biofilm development [8,15].

Research indicated a correlation between the abundance of *E. coli* in the stomach and bladder during recurrent UTI episodes [16], suggesting a connection between these populations in both locations. This element can further clarify the effectiveness of EcN in preventing rUTIs, drawing on the extensive literature regarding its use for intestinal issues. This understanding may pave the way for new treatment protocols that incorporate EcN as a preventative measure for rUTIs, potentially reducing the reliance on antibiotics and enhancing patient outcomes.

EcN has demonstrated efficacy in competing with *E. coli* and other enteropathogens, and it seems to be a safe therapeutic treatment that does not generate enterotoxins or cytotoxins linked to pathogenic *E. coli* strains. Furthermore, no differences were seen in the intestinal tissue analysis between treated and control rats, indicating that EcN did not demonstrate any genotoxic activity [17].

This study has several limitations that should be acknowledged. First, the relatively small sample size and single-center design may limit the generalizability of our findings; in addition, the follow-up period of six months, although sufficient to assess short-term recurrence, may not capture long-term outcomes or seasonal variations in UTI incidence. Moreover, the open-label design without placebo control may have introduced performance bias, although this risk was mitigated by the use of urine culture to confirm recurrence rather than relying solely on LUTS onset. Further research is needed to address these limitations and to validate the findings across larger, more diverse populations. Future studies should also consider incorporating a placebo group to strengthen the evidence for EcN’s effectiveness.

## 5. Conclusions

Individuals with rUTI require thorough assessment to optimize treatment strategies and minimize unnecessary antibiotic use. Management may encompass both antibiotic and non-antibiotic prophylactic strategies. Nonetheless, we underscore the significance of investigating non-antibiotic prophylactic alternatives such as EcN. Our prospective trial demonstrated that prophylaxis with *E. coli* Nissle 1917 (EcN) was effective in reducing the recurrence of urinary tract infections, with a significantly higher proportion of UTI-free patients compared with the untreated control group after six months of follow-up.

Future research should aim to identify patient subgroups that may benefit most from this intervention. Such studies should incorporate microbiome-focused sub-analyses to elucidate the mechanisms underlying EcN’s protective effects and to identify patient-specific microbial signatures predictive of treatment response. In addition, long-term studies with larger cohorts are warranted to confirm these findings and assess the sustainability of EcN prophylaxis over time. Moreover, comparative trials between EcN and other non-antibiotic strategies could contribute to defining tailored, antibiotic-sparing prevention strategies for rUTI.

## Figures and Tables

**Table 1:** Baseline Characteristics of the Study Population.

Baseline Characteristics	EcN Group = 20	Control Group = 20	*p*-Value
Age, years (range)	34.9 (20.0–43.0)	38.1 (23.0–44.0)	NS
BMI, kg/m^2^ (range)	24.2 (19.1–26.2)	23.8 (18.0–25.1)	NS
Smoking habits *n* (%)	11 (55)	10 (50)	NS
Length of menstrual cycles			
Regular (21–35 days)	15 (75)	16 (80)	NS
Oligomenorrhea (>35 days)	5 (25)	4 (20)	
OC use			
No	15 (75)	13 (65)	NS
SI	5 (25)	7 (35)	
Parity			
Nulliparous	18 (90)	17 (85)	NS
Multiparous	2 (10)	3 (25)	
Sexually active in the past 3 months, *n* (%)	13 (65)	14 (70)	NS

Continuous data are expressed as mean (range) and categorical data are expressed as absolute numbers (percentages). Mann–Whitney U test for continuous data, Fisher test for categorical data. OC = Hormonal contraceptives. mean (SD). NS = not significant.

## Data Availability

The data presented in this study are available on request from the corresponding author due to privacy.

## Abbreviations

The following abbreviations are used in this manuscript:

UTI	Urinary tract infection
rUTI	Recurrent urinary tract infection
UPEC	Uropathogenic *E. coli*
EcN	*Escherichia coli* Nissle 1917
CFUs	Colony-forming units
LUTS	Lower urinary tract symptom
BMI	Body mass index
